# Research on neurophysiological and behavioral measures of attentional and inhibitory processes in adult young with ADHD

**DOI:** 10.1192/j.eurpsy.2021.1960

**Published:** 2021-08-13

**Authors:** L.R. Carreiro, A.A. Osório, M.C. Teixeira, W. Machado-Pinheiro, J. Nascimento, A. Afonso Junior

**Affiliations:** 1 Developmental Disorders Graduate Program, Mackenzie Presbyterian University, São Paulo, Brazil; 2 Departamento De Ciências Da Natureza, Universidade Federal Fluminense, Rio das Ostras, Brazil

**Keywords:** ADHD, temporal and spatial attention, fNIRS, inhibition

## Abstract

**Introduction:**

Attention Deficit Hyperactivity Disorder (ADHD) is characterized by harmful levels of inattention, hyperactivity, and impulsivity and occurs in 2.5% of adults.

**Objectives:**

This project will evaluate young adults with ADHD in computerized tasks that assess different forms of attention and inhibition, correlating them with self-report scales and physiological measurements (EMG and fNIRS) to identify impairments in specific cognitive domains.

**Methods:**

The study will be conducted with two groups: one with ADHD - GClin and one control - CG, with 50 participants between 18 and 28 years each. Initially, participants will perform CPT-3 and respond to ASRS to be allocated to the CG or GClin, with validation by a specialist physician. After that, they will do the computerized inhibition (Stroop / Stop) temporal and spatial attention (voluntary and automatic) tests. In this phase the data will be collected using electromyographic measurements and recording of brain activity in areas of the prefrontal and temporal cortices through fNIRS. After the tests they will complete the impulsivity scales (BIS-11 and UPPS). The analyzes will comprise: (1) ANOVA of the means of TRs and the accuracy of the computerized tests; (2) Correlation analysis of RT, accuracy and ASRS scores; and (3) The fNIRS analysis will use the oxyhemoglobin signal, which will be analyzed individually.

**Results:**

As expected results there will be differences between CG or GClin in relation to impulsiveness, number of errors and brain activation.

**Conclusions:**

The integration of physiological measurements, scales and tests will ensure integrated understanding of attentional and inhibitory processes impaired in ADHD.

**Disclosure:**

No significant relationships.

